# The Learning Curve in neurofeedback of Peter Van Deusen: A review article

**DOI:** 10.1590/S1980-5764-2016DN1002005

**Published:** 2016

**Authors:** Valdenilson Ribeiro Ribas, Renata de Melo Guerra Ribas, Hugo André de Lima Martins

**Affiliations:** 1 Graduated in Psychology, Expert in Mental Health, Masters and Doctorate in Neuropsychiatry, UFPE;; 2 Graduated in Nutrition, Expert in Phytotherapy;; 3 Graduated in Medicine, Expert in Neurology and Psychiatry, Masters and Doctorate in Neuropsychiatry, UFPE.

**Keywords:** brain-learning, learning curve, neurofeedback

## Abstract

The Learning Curve (TLC) in neurofeedback concept emerged after Peter Van Deusen compiled the results of articles on the expected electrical activity of the brain. This concept was subsequently tested on patients at four clinics in Atlanta between 1994 and 2001. The aim of this paper was to report the historical aspects of TLC. Articles published on the electronic databases MEDLINE/PubMed and Web of Science were reviewed. During patient evaluation, TLC investigates categories called disconnected, hot temporal lobes, reversal of alpha and beta waves, blocking, locking, and filtering or processing. This enables neuroscientists to use their training designs and, by means of behavioral psychology, to work on neuroregulation, as self-regulation for patients. TLC shows the relationships between electrical, mental and behavioral activity in patients. It also identifies details of patterns that can assist physicians in their choice of treatment.

## RESUMO

A TLC em *neurofeedback* surgiu após uma reunião de periódicos organizada por Peter Van Deusen sobre as atividades elétricas cerebrais esperadas e depois testadas em diversos pacientes em quatro consultórios, em Atlanta, de 1994 a 2001. O objetivo deste artigo é relatar o aspecto histórico da TLC. Realizou-se uma revisão na base eletrônica MEDLINE/PubMed e *Web of Science*. A TLC investiga as categorias denominadas desconectados, temporais quentes, inversões de alpha e beta, bloqueando, trancando, filtrando e processando e, em seguida, possibilita, em seus designs de treinamento, que o (a) neurocientista trabalhe, por meio da psicologia comportamentalista, a autoneuroregulação do paciente. A TLC mostra as relações entre as atividades elétricas, mentais e comportamentais nos pacientes e também fornece uma identificação detalhada dos padrões que podem ajudar os médicos na escolha dos tratamentos.

## INTRODUCTION

### Electroencephalography (EEG): a brief history 

Electricity was first described by Thales of Miletus (624-546 BC)^1^ when he observed that lodestone (magnetite), a naturally occurring magnet, and amber (after rubbing with fur) attracted other objects. Later, William Gilbert (1544-1603)^2^ published a study showing the Earth was magnetic and that this was why compasses point North; Otto von Guericke (1602-1686)^3^ invented an electrostatic machine consisting of a sulfur sphere; Pieter van Musschenbroek (1692-1761)^4^ became renowned in the field for creating the Leyden jar, a device to store electrical charge. A number of scholars furthered our understanding of the electrical properties of living organisms, including Luigi Galvani (1737-1798),^5^ Alessandro Volta (1745-1827),^6^ Georg Ohm (1789-1854)^7^ and Michael Faraday (1791-1867).^8^ In around 1780, for example, Galvani dissected a frog and left it on a lab bench. However, the frog twitched when one of his assistants touched its crural nerve with a scalpel.^9^ It was at this time that Galvani began to establish a relationship between electrical activity and nerve impulses.^10^ ^,^ ^11^

A contemporary of Galvani, Alessandro Giuseppe Antonio Anastasio Volta (1745-1827),^12^ became intrigued with Galvani's ideas; however, perhaps because he had graduated in physics, he believed that living organisms could not actually produce electricity.^13^ Subsequently, in Florence, Leopoldo Nobili (1784-1835)^14^ invented the needle galvanometer. This galvanometer was refined in 1858 by William Thomson (1824-1907),^15^ Carlo Matteucci (1811-1868)^16^ and particularly the German researcher, physiologist Emil du Bois-Reymond (1818-1896)^17^ who demonstrated nerve action potential.^18^ In 1875, Richard Caton (1842-1926)^19^ described the electrical brain activity of rabbits and monkeys, using a one-channel galvanometer. This experiment marked the birth of electrophysiology.^11^ ^,^ ^20^

The difficulty at that time was to find sophisticated equipment that could capture the low-intensity electrical activity of the brain (microvolts). In this scenario, another researcher named Willem Einthoven (1860-1927)^21^ excelled by improving galvanometers and allowing their use in cardiology and neurology, for which he received the Nobel prize for medicine in 1912.^11^ ^,^ ^21^

Later, a researcher called Vladimir Pravidich-Neminsky (1879-1952)^11^ ^,^ ^22^ captured the electrical activity of a dog's nerves using an Einthoven Galvanometer, recording the image on photographic paper adhered to a revolving drum. The results, waves shown as traces over time, were published in 1913 and recognized as the first printed electroencephalogram (EEG).^22^ At around this time, Hans Berger (1873-1941)^23^ was the first scientist to record human brain electrical activity using the Einthoven Galvanometer. It was Berger who described alpha and beta rhythms and proposed the EEG model of 3 cm per second still used today.^24^ Edgar Douglas Adrian (1889-1977)^25^ reproduced and published several studies based on Berger's findings.^26^ In 1935, in the United States, Herbert Henri Jasper (1906-1999),^27^ using more modern and sensitive equipment than Berger's devices, published many studies on animals and humans.^11^ ^,^ ^27^

Electroencephalography developed further with the studies on epilepsy by the Gibbs couple, Frederic Andrews Gibbs (1903-1992) and Erna L. Gibbs (1925-1987),^28^ who also described the complex spike-wave.^11^ ^,^ ^29^ The first multi-channel devices were created by Albert Melvin Grass (1910-1992)^30^ in 1939. Subsequently, Nathaniel Kleitman (1895-1999),^31^ William Charles Dement (1928),^32^ Giuseppe Moruzzi (1910-1986)^33^ and Horace Winchell Magoun (1907-1991)^34^ contributed much to electroencephalography with their discoveries and descriptions of the stages of sleep and the K-complexes.^11^ ^,^ ^35^

After the discovery of qualitative EEG by Berger, the physicist Dietsch (1932) from the Physics and Technology Institute at Jena, Germany, applied Fourier analysis to seven EEG recordings and became the first researcher in quantitative EEG (QEEG).^36^ This achievement, in conjunction with the advancement of computing and digital EEG, enabled the discovery of Biofeedback/Neurofeedback.^37^

## EEG-BIOFEEDBACK/NEUROFEEDBACK: A PRACTICAL APPLICATION

Concurrent with the development of the digital EEG for assessment purposes, a separate group of researchers began demonstrating the capacity of the brain to shift its own activation patterns when given feedback. In 1968, Dr. Joe Kamiya published an article about his experiences with alpha brain waves in Psychology Today.^38^ Despite criticism by Martin Orne and others, Kamiya and James Hardt at the Langley Porter Psychiatric Institute of the University of California published an original article demonstrating the effectiveness of training by neurofeedback.^39^

Although the Fast Fourier Transform (FFT) had been used to break down and digitize the analog waveforms of the traditional EEG signal, the process was very time-consuming until the advent of the digital computer in the 1970s.^37^ Increasingly, as computation grew in speed and power while becoming more accessible in size and cost over the ensuing decades, digital quantitative EEG has moved from large university laboratories^37^ ^,^ ^40^ literally into the offices of individual professionals.^37^ ^,^ ^41^ Because of its high test/retest reliability and temporal resolution, it has formed the basis for much of the research that, during the 1990s, provided a detailed view of the living brain that infuses the "geographical" brain of Brodmann and others.^36^ ^,^ ^37^

In the 1980s, researchers such as E. Roy John^42^ ^,^ ^43^ and Robert Thatcher^44^ ^,^ ^45^ began aggregating processed EEGs into so-called "normative databases".^44^ ^,^ ^46^ ^,^ ^47^ These quickly became an integral part of the QEEG used by brain researchers, allowing them to compare EEG patterns of sub-groups of the population (e.g. anxious, compulsive, inattentive, etc.) against the population as a whole and to identify differentiating variables.^46^

## HISTORY OF THE TLC ASSESSMENT (TRAINERS' QEEG)

In 1991, Peter Van Deusen formed a mentoring relationship with Joel F. Lubar, a professor at the University of Tennessee-one of the pioneers and most prolific researchers in the field.^48^ After training his own brain and experiencing significant changes, he had an opportunity to begin working under the supervision of two psychologists-a behaviorist and a family therapist-and a psychiatrist/psychopharmacologist. Integrating these three apparently disparate viewpoints for nearly a year led to a view of the process that would inform and underlie the system he developed for brain training.^49^

Following that experience, Van Deusen continued with the project of applying the technology of brain training, not from a pathology-based viewpoint but as a means of producing lasting changes in the patterns and systems that presented at his practice on Attention Development Programs (ADP).^50^

He argued that mental health diagnoses, which were proliferating at a dizzying pace during the 1990s, were largely just descriptions of symptoms, so his approach focused on identifying behavioral issues targeted for change without labeling them. In courses with various colleagues breaking new ground in the field of brain training during this period, he recognized multiple descriptors of brain function, including patterns and relationships of frequencies, synchrony, symmetry and variability that could identify macro divergences from the peak brain patterns that most interested him.^49^

From 1993 to 2001, ADP operated 3-4 practice sites in Atlanta and worked with more than 500 patients with a broad range of training issues. Van Deusen did all assessments and training plans, personally trained nearly 200 patients in one center and supervised the trainers in the others.^49^ During this period, he implemented multiple training approaches as the technology advanced. In 2001, Van Deusen left his practice and began traveling half or more of each year to teach courses on assessment and training to professionals in 32 US states, Canada, Australia, Korea, Brazil and six European countries.^48^ During this period, he began gathering material from a number of published studies identifying patterns in the EEG and formalizing them into a brain-based assessment process, which he presented at various national conferences. This TLC Assessment^51^ began being used by professionals around the world.^49^

## TLC TRAINING CATEGORIES

The Learning Curve (TLC) in neurofeedback is a brain training technique based on an investigative protocol developed by Peter M. Van Deusen,^49^ ^,^ ^51^ following compilation of results of articles about expected patterns of brain electrical activity in humans. According to Sterman et al. (1996),^52^ changes in patterns that involve the predominance of expected waves and the synchronization between the two cerebral hemispheres may represent different cognitive and behavioral states, including signs and symptoms of attention deficit, depression, anxiety and fear, among others.^49^ ^,^ ^53^

The TLC protocol^49^ ^,^ ^51^ was created from reading, interpreting and reproducing the results of a number of authors.^54^ ^-^ ^58^ Van Deusen combined six categories, classified by the authors cited above,^54^ ^-^ ^58^ in the protocol to be used by all trainers that employ the TLC method. These are: 1) disconnected; 2) hot temporal lobes; 3) reversals; 4) blocking; 5) locking; 6) filtering or processing. 

Disconnected is a category that emerged from reading several studies by Martin Teicher et al.^54^ ^-^ ^60^ Teicher investigated the effects of physical and psychological abuse in childhood, and demonstrated neurological morphological, functional and behavioral abnormalities in the frontal and temporal lobes. These changes possibly derive from effects caused by the promoter scenario of anger, shame and desperation of the victim.

Thus, Van Deusen started to observe the patients that he treated in Atlanta and found, during the workup, several reports involving situations of physical and psychological abuse and negligence. One individual was not physically abused as such, but reported that all her sisters had been sexually abused by her father. She then added that she was his 'darling' and suffered a constant invasion of personal limits that had a similar nervous system response. With much distress, this victim reported that "every day when I woke up, I saw my father sitting on my bed staring at me". She reported that over time, this situation became so unbearable that she started waking up earlier, before her father came to her room, and hiding under the bed.^49^

After this case, between 1994 and 2001, Van Deusen reread articles on cases of abuse that generally were of a psychological nature and realized that there are nuances of psychological abuse at the conceptual level, involving the paradox between very intense religious beliefs and breaking these dogmas.^48^ ^,^ ^49^ The results of Van Deusen using QEEG identified a pattern related to high beta waves (23-38 Hz) with the patient's eyes closed. All the results in the right temporal lobe (T4), both percentages and relative numbers, were exactly double those in the left temporal lobe (T3). However, the result was exactly the opposite (T3 = 2T4) in cases of neglect, regardless of whether it was an intentional separation (conscious rejection) or a necessity due to a disease that forced the mother to stay away from her baby.^49^ ^,^ ^57^ ^,^ ^59^

Hot temporal lobe is a category that emerged from reading several studies by Othmer and other authors,^49^ ^,^ ^61^ ^-^ ^65^ involving discussions about the activation of the amygdalae in the temporal lobes that use the beta and high beta waves to communicate with the hypothalamus to warn of danger. Peter M. Van Deusen et al. observed patients who had anxiety, panic, fear, phobia or insecurity and found a pattern of waves in the temporal lobes with their eyes closed, for both (T3) and (T4); the beta waves (15-23 Hz) were above 17% and high beta waves above 10%. An ideal pattern of expected beta waves of 14-17% and high beta waves of at most 10% were found by studying a control group without any of these feelings.^49^ ^,^ ^62^

Reversal is a category based on the findings of authors^51^ ^,^ ^56^ ^,^ ^61^ ^,^ ^63^ ^,^ ^66^ ^,^ ^67^ who prompted Van Deusen to study and understand the patterns of alpha and beta waves. Van Deusen and his team confirmed that the right hemisphere (Fp2, F4, F8, C4 and T4) and rear part of the brain (P4, T6 and O2) are optimal regions for an abundance of alpha waves.^51^ ^,^ ^68^ Thus, alpha waves are expected to be higher (10-15%) in the right side and posterior brain compared to the left side and anterior brain. According to these findings, when there is a reversal of alpha waves, the person may experience symptoms of depression with hopelessness (helpless, sad, with little energy and some suicidal ideations). On the other hand, a predominance of beta waves is expected in the front and left side of the brain (5%) and, if there is a reversal, the individual may experience symptoms of agitated depression (moody, angry, irritable, nervous and/or anxious).^51^

Van Deusen and his team corroborated other findings^51^ ^,^ ^56^ ^,^ ^61^ ^,^ ^63^ ^,^ ^66^ ^-^ ^68^ and described emotional features of the prefrontal cortex involving both the left and right hemispheres. Consequently, the left hemisphere was named the side of approximation, presenting characteristics of happiness, expansiveness, enthusiasm and optimism.^51^ The right hemisphere, on the other hand, was called the side of avoidance, demonstrating dark, depressed, and withdrawal feelings and rage.^49^ Thus, it is important to maintain a balance (left/right or manic versus depressive), with the left side being slightly more dominant as it processes around 5% more beta waves (15-23 Hz).^49^ ^,^ ^51^ Similarly, these authors analyzed the anterior and posterior portions of the brain and stated that the alpha waves should predominate in the back of the brain because they are an electrical brain activity that serves to integrate data, while beta waves should predominate in the front of the brain, as their function is to process data and take action.^51^ ^,^ ^61^ ^,^ ^66^

Blocking is a category based on the experiences of Amen et al.,^69^ ^-^ ^72^ who explained the function of the anterior cingulate cortex and proposed the term 'hot cingulate'.^73^ ^,^ ^74^ This expression is used for the set of symptoms caused by impaired communication between the emotional circuitry that surrounds nerve cells of the orbitofrontal region, basal ganglia and anterior cingulate gyrus, which is responsible for emotional responses to pain, regulation of aggression and emotions, motivation, resolution of cognitive conflict, and completing tasks.^71^ ^,^ ^75^ When conflict arises in communication between the emotional circuitry and anterior cingulate gyrus, symptoms arise such as obsessive thoughts, compulsive behavior, addictions and/or phobias. There is a mixture of emotion and reason in thought processes. Obsessive Compulsive Disorder (OCD) is an example of dysfunction of the anterior cingulate cortex.^49^

Locking is a category based on the findings of authors dedicated to studying the communication between the brain hemispheres.^76^ ^-^ ^79^ Their studies motivated clinical studies of Van Deusen observing there are norms for the percentage of coherence between slow and fast waves. Thus, between 1994 and 2001, Peter M Van Deusen observed and confirmed patterns, as very low coherence is related to little cooperation between the areas and consequently reduced efficiency, slow processing and errors.^78^ Low synchrony levels can be related to injuries or physical disturbances in transmission, but are often the result of overly excitable brains, which burst into beta when there is no task to be done, thus blocking the resonance. Very high consistency characterizes excess communication between the left hemisphere (predominance of reason) with the right hemisphere (predominance of emotion), unnecessary communication between the areas with locked responses, which can be related to mental rigidity or obsessiveness, perhaps to anxiety, inflexibility and diminished creativity.^79^

Filtering or processing is based on the ratio of the percentage of theta waves divided by beta waves, and expresses the relationship between rational and intuitive styles of thought of an individual. When the ratio of theta (4-8 Hz) to Beta (13-21 Hz) exceeds 2 in an adult with eyes open, cognitive processing issues begin to appear and increase as the ratio grows. These issues are identified in a category called "Processing". Ratios below 1.2 in adults correlate with distractible/impulsive styles often related with hyperactivity. The category for this pattern is Filtering.^50^ ^,^ ^80^ ^,^ ^81^ The original data were gathered at Cz in the sensorimotor cortex, but the TLC extends it as a general indicator for all sites.^49^ ^-^ ^51^

The alpha/theta ratio metaphorically represents a bridge between subconscious and conscious processes. Thus, a person can know that he/she is angry and why he/she is angry or can be angry and not understand why he/she is angry.^82^

The expected value for the ratio of the total percentage of alpha waves divided by the total percentage of theta waves is 1.0 in the frontal cortex when the individual has his eyes closed. When the eyes are open, this value should decrease to 0.7 and, if the individual is doing a task, this value should remain at 0.7 or drop further; 1.5 for the back of the cortex when the individual is with his eyes closed. When he/she opens his eyes, it is expected that this value should decrease to 0.9 and, if the individual is doing some task, the value is expected to remain at 0.9 or decrease even further; the central portion of the cortex (sensorimotor) can follow the same patterns as the frontal cortex or posterior cortex.^82^ ^,^ ^83^
